# Melatonin for rapid eye movement sleep behavior disorder in Parkinson's disease: A randomised controlled trial

**DOI:** 10.1002/mds.27886

**Published:** 2019-10-31

**Authors:** Moran Gilat, Alessandra Coeytaux Jackson, Nathaniel S. Marshall, Deborah Hammond, Anna E. Mullins, Julie M. Hall, Bernard A.M. Fang, Brendon J. Yee, Keith K.H. Wong, Ron R. Grunstein, Simon J. G. Lewis

**Affiliations:** ^1^ Woolcock Institute of Medical Research, The University of Sydney Sydney Australia; ^2^ ForeFront Parkinson's Disease Research Clinic Brain and Mind Centre, The University of Sydney Sydney Australia; ^3^ Research Group for Neurorehabilitation (eNRGy), Department of Rehabilitation Sciences, KU Leuven Leuven Belgium; ^4^ Department of Neurology University Hospitals of Geneva Switzerland; ^5^ Department of Respiratory and Sleep Medicine Royal Prince Alfred Hospital Sydney Australia

**Keywords:** melatonin, Parkinson's disease, randomized controlled trial, REM sleep behavior disorder, sleep disorders

## Abstract

**Background:**

Melatonin may reduce REM‐sleep behavior disorder (RBD) symptoms in Parkinson's disease (PD), though robust clinical trials are lacking.

**Objective:**

To assess the efficacy of prolonged‐release (PR) melatonin for RBD in PD.

**Methods:**

Randomized, double‐blind, placebo‐controlled, parallel‐group trial with an 8‐week intervention and 4‐week observation pre‐ and postintervention (ACTRN12613000648729). Thirty PD patients with rapid eye movement sleep behavior disorder were randomized to 4 mg of prolonged‐release melatonin (Circadin) or matched placebo, ingested orally once‐daily before bedtime. Primary outcome was the aggregate of rapid eye movement sleep behavior disorder incidents averaged over weeks 5 to 8 of treatment captured by a weekly diary. Data were included in a mixed‐model analysis of variance (n = 15 per group).

**Results:**

No differences between groups at the primary endpoint (3.4 events/week melatonin vs. 3.6 placebo; difference, 0.2; 95% confidence interval = −3.2 to 3.6; *P* = 0.92). Adverse events included mild headaches, fatigue, and morning sleepiness (n = 4 melatonin; n = 5 placebo).

**Conclusion:**

Prolonged‐release melatonin 4 mg did not reduce rapid eye movement sleep behavior disorder in PD. © 2019 The Authors. *Movement Disorders* published by Wiley Periodicals, Inc. on behalf of International Parkinson and Movement Disorder Society.

Rapid eye movement (REM) sleep behavior disorder (RBD) causes a loss of muscle atonia during REM sleep, leading to dream enactment behaviors that are frequently injurious to patients and their partners.^1^ Its prevalence in adults is around 1%,^1^ compared to 20% to 50% of people with Parkinson's disease (PD).^2^, ^3^

Treatment includes clonazepam or melatonin.^3^, ^4^ Clonazepam is a long‐acting benzodiazepine linked to adverse outcomes,^3^, ^4^ whereas its efficacy for reducing RBD in PD was recently challenged.^5^ Melatonin has a safer profile with milder side effects, such as headache and morning sleepiness.^2^, ^4^, ^6^, ^7^, ^8^, ^9^ Melatonin has a 30‐ to 50‐minute elimination half‐life.^10^ A prolonged‐release (PR) formulation has thus been recommended for RBD, given that most REM sleep occurs later in the night.^11^

However, current guidelines are based on mainly case series and small open‐label studies.^6^, ^7^, ^9^, ^11^, ^12^, ^13^, ^14^, ^15^, ^16^ Only one previous randomized controlled trial (RCT) assessed the efficacy of 3 mg of melatonin in just 8 subjects with mixed neurological disorders, showing a modest improvement on the clinical global impression (CGI) and REM epochs without atonia.^8^ However, another small RCT recently showed that 2 (n = 7) or 6 mg (n = 9) of PR melatonin did not improve CGI versus placebo (n = 9) in subjects with clinically isolated RBD.^17^ No RCT has been conducted for RBD in PD. Moreover, there is need of an RBD‐specific outcome that is free from subjective interpretation by study assessors.^4^

We set out to assess the efficacy of 4 mg of PR melatonin on RBD severity using patient‐reported diary entries. We hypothesized that melatonin would reduce RBD compared to placebo.

## Materials and Methods

### Design

A phase‐II, randomized, double‐blind, placebo‐controlled, parallel‐group trial with an 8‐week intervention and 4 weeks of observation pre‐ and postintervention (Supporting Information Fig. S1) was conducted at the Woolcock Institute of Medical Research (Sydney, Australia). The pre‐observation weeks served for screening eligibility. The post‐observation period assessed whether the effects would persist.^8^, ^11^ Ethical approval was obtained from the Human Research Ethics Committees of the University of Sydney and Royal Prince Alfred Hospital, Australia. The trial was prospectively registered (ACTRN12613000648729) with its protocol published online: http://www.anzctr.org.au/ACTRN12613000648729.aspx.

### Participants

We set out to recruit 30 PD participants with video‐ polysomnography (PSG)‐confirmed RBD. Supporting Information Table S1 lists the eligibility criteria. All participants gave written informed consent in accordance with the Declaration of Helsinki.^18^

### Randomization

Participants were randomized (1:1) using a computerized blocked randomization sequence by the trial epidemiologist (N.S.M.) not involved with recruitment or assessment. The placebo was identical in appearance to the melatonin. Independent pharmacists prepared identical treatment bottles based on the randomization sequence that remained concealed to all other staff. Randomization was performed by providing participants with their respective bottles, which contained the exact amount of study drug required. Masking was thereby ensured for patients and staff giving the intervention, assessing the outcomes, and analyzing the data.

### Procedures

The intervention consisted of 4 mg (1 × 2 tables‐2 mg) of PR melatonin (Circadin) or 4 mg (1 × 2 tables‐2 mg) of matched placebo (lactose‐monohydrate) produced by Neurim Pharmaceuticals Inc. (Tel Aviv‐Yafo, Israel). Participants were instructed to ingest the trial drug orally, once‐daily, after food, within 1 hour before bedtime, for 8 weeks.

Participants completed the weekly CIRUS‐RBD Questionnaire (wCIRUS‐RBDQ; Supporting Information), every morning about the preceding night. They were instructed to record any instance of RBD as noted by themselves and/or their partners (if applicable).

A video‐PSG was performed within 12 months before randomization and within weeks 5 to 8 of the treatment period.

Participants wore a Philips Respironics Actiwatch 2 (Koninklijke Philips N.V., Amsterdam, Netherlands) on the wrist least affected by PD^19^ and filled out an associated diary for 1 week during baseline and again within weeks 5 to 8 of treatment. Actigraphy was scored manually using Philips Respironics Actiware‐5 software (Koninklijke Philips N.V.).

The protocol included five monthly visits (Supporting Information Fig. S1). Participants completed the RBD Screening Questionnaire, Innsbruck RBD Inventory, and RBD‐Questionnaire Hong Kong, at visits 1, 2, 4, and 5. Secondary questionnaire outcomes (Supporting Information Table S2) were completed at baseline and weeks 5 to 8 of treatment. During visit 2, final eligibility was assessed, followed by the CGI and International Parkinson and Movement Disorder Society (MDS)‐UPDRS. Eligible participants were randomized at visit 2. During visit 3, a safety assessment was conducted, and during visit 4, another CGI and MDS‐UPDRS were performed. During visit 5, a final CGI was conducted.

### Outcomes

The primary outcome was the difference in mean total number of RBD events captured by the wCIRUS‐RBDQ item‐4. The primary endpoint was taken as the aggregate of all RBD incidents averaged over weeks 5 to 8 of treatment. All other measures are regarded secondary in importance (Supporting Information Table S2). A medical officer assessed adverse events at each visit.

### Analysis

Sample size was arbitrarily determined by our budget and because no studies could be used to calculate sample size using our novel primary outcome. Assuming a 7% dropout, 28 patients would complete the trial. We calculated that we would be 90% powered (α = 5%) to detect a drop from an assumed 2.25 to 1.0 RBD events per week (56% reduction) with an assumed standard deviation of 1.0 on the primary outcome. Given that only 1 subject dropped out, this study was powered to detect the preset change in RBD symptoms. Patients were analyzed in the group they were randomized to. There were no interim analyses, and the trial stopped when we recruited our intended sample size.

The primary and secondary outcomes derived from the wCIRUS‐RBDQ were analyzed by mixed‐model analyses of variance in SAS software (version 9.4; SAS Institute Inc., Cary, NC) using the patient as random effects and drug randomization and time (weeks into the trial) as fixed effects. The primary endpoint was compared between groups with a least means square test within the interaction between time and treatment (regardless of the main interactions). The model used all interim measures to reduce measurement error within patients. Subjects were categorized as treatment responders (CGI = 1–3) or nonresponders (CGI ≥4) at visit 4. The proportion of responders was compared across groups using Fisher's exact test. For all other outcomes, the delta between visits 4 and 2 was computed and compared between groups using two‐sided *t* tests or the Mann‐Whitney U test using IBM‐SPSS software (version 25.0; IBM Corp., Armonk, NY) with an alpha of 5%.

A post‐hoc analysis was performed to assess whether the outcomes were influenced by variations in bedtimes (Supporting Information).^8^, ^20^ Adherence in completing the wCIRUS‐RBDQ was determined by the percentage of missing entries over the total amount of entries expected.

## Results

### Participants

**Figure 1:**
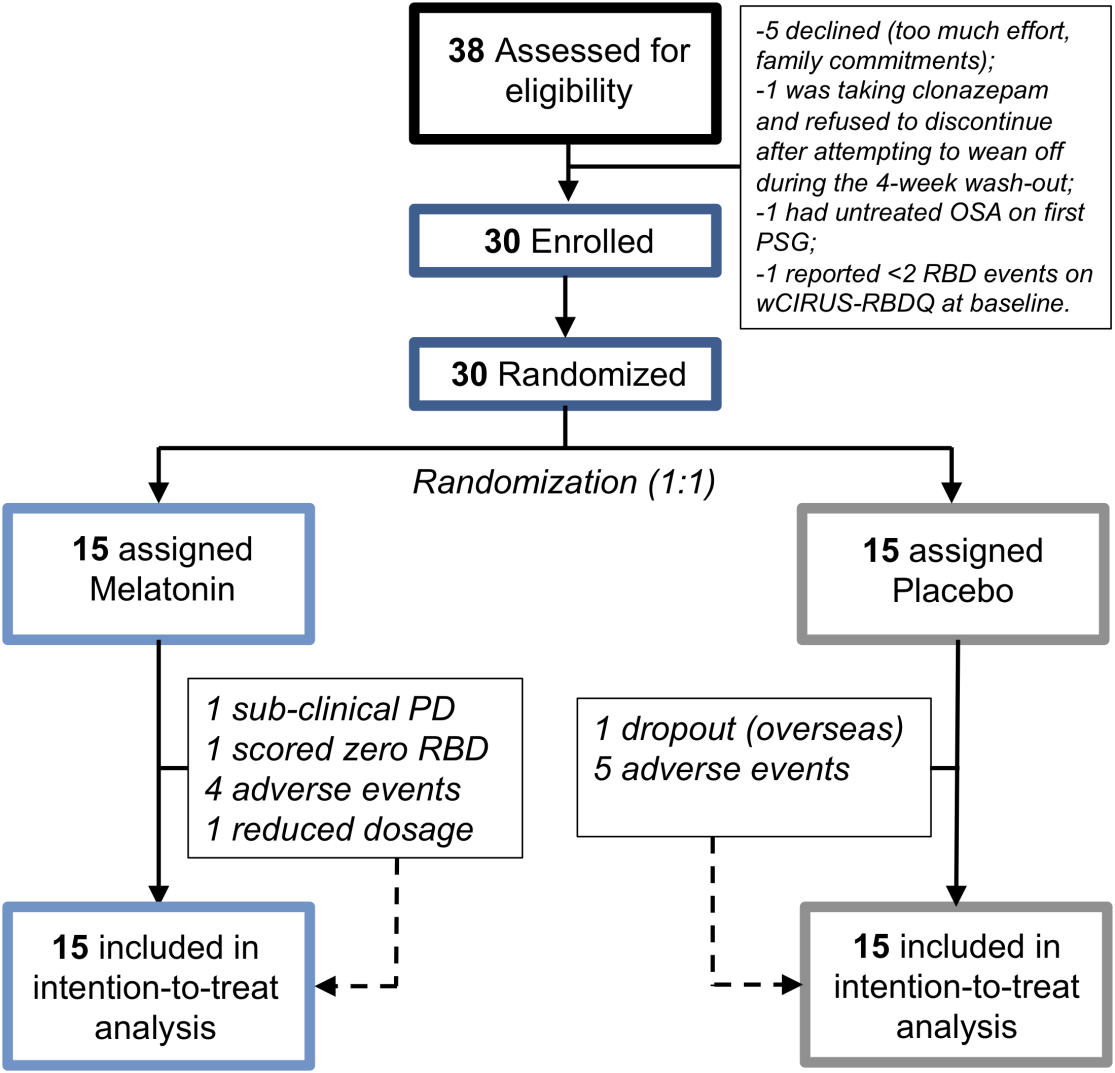
Trial profile. [Color figure can be viewed at http://wileyonlinelibrary.com]

Thirty‐eight participants were screened between August 1, 2013 and October 5, 2017. Eight were deemed ineligible (Fig. 1). The remaining participants were randomized to melatonin (n = 15) or placebo (n = 15). One participant on placebo dropped out in the first month after moving overseas. Two protocol violations occurred whereby 2 participants with clinical and video‐PSG‐confirmed RBD were randomized into the melatonin group who should not have been. One participant who 3 months after the study developed PD had subclinical MDS diagnostic criteria at baseline,^21^ and another scored zero RBD events on the wCIRUS‐RBDQ. All data were entered into the primary analysis based on an intention to treat. Participants’ baseline characteristics are reported in Supporting Information Table S3.

### Outcomes

**Figure 2:**
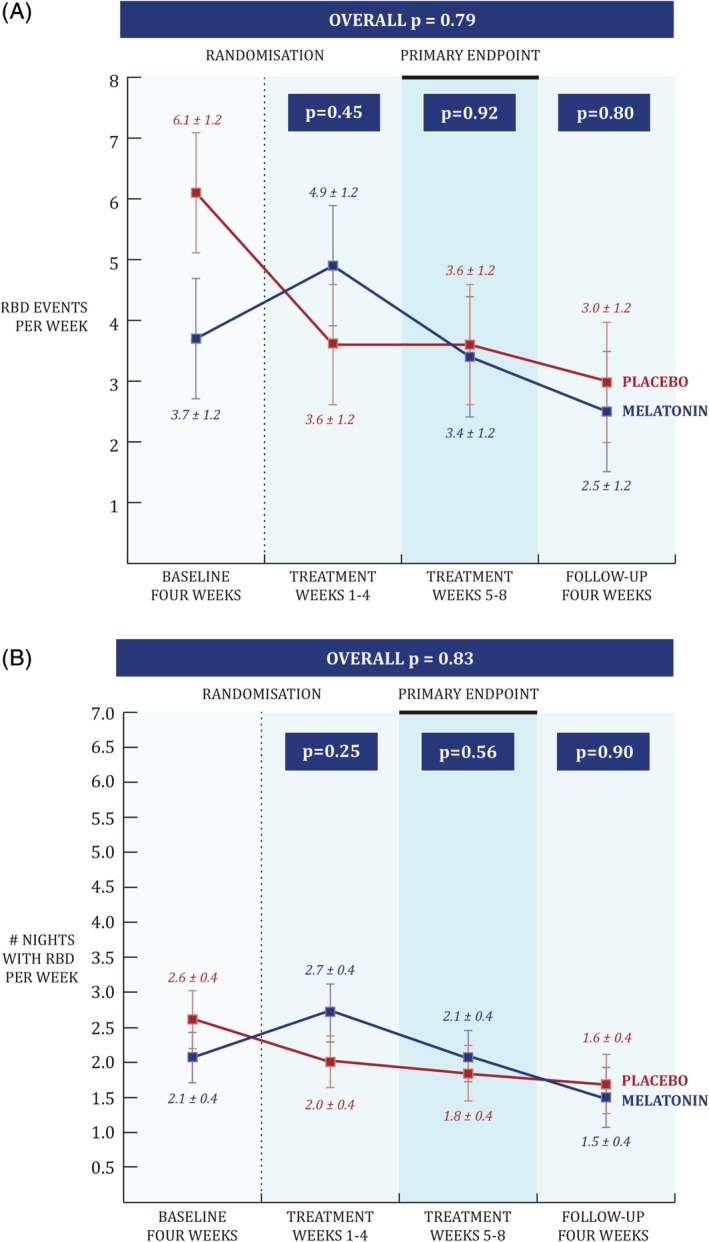
Primary and secondary wCIRUS‐RBDQ outcome. Patient and partner reported dream enactment events were collected every day in weeklong diary reports and then averaged in 4‐week epochs that represent the four phases of the study (pretreatment, early and late treatment, and follow‐up). The primary endpoint of the study was the late treatment epoch (weeks 5–8). Panel (**A**) shows that there was no significant difference between melatonin (in blue) and placebo (in red) on the number of RBD events (primary outcome; *P* for difference = 0.92). The absolute range of RBD events per week underlying the data at baseline were 0 to 28 (28 being an outlier) for the melatonin group and 0 to 18 events per week for the placebo group. Panel (**B**) shows that there was no significant difference between groups on the number of nights in which a dream enactment event occurred RBD events (secondary outcome 1; *P* for difference = 0.56). The error bars drawn are the standard errors. [Color figure can be viewed at http://wileyonlinelibrary.com]

No reduction in RBD was found between groups (3.4 events/week melatonin vs. 3.6 placebo; absolute difference: 0.2; 95% confidence interval [CI] = –3.2 to 3.6; *P* = 0.92, Fig. 2A). Secondary outcomes also revealed no difference for the number of nights in which RBD was reported (2.1 nights/week melatonin vs. 1.8 placebo; difference, 0.35; 95% CI = –0.8 to 1.5; *P* = 0.56, Fig. 2B) or change in the frequency of vivid dreams (2.4 dreams/week melatonin vs. 2.9 placebo; difference, –0.6; 95% CI = –2.2 to 1.1; *P* = 0.49). RBD‐related injuries could not be analyzed, because only 5 participants (3 melatonin, 2 placebo) reported an injury. Sensitivity analysis without the participant in the melatonin group who scored zero RBD events did not change our conclusions (*P* = 0.97; see Supporting Information).

The average sleep‐onset latency on actigraphy decreased with melatonin compared to placebo (U = 12.0; Z = –3.17; *P* = 0.002; ŗ = 0.68). The 36‐item Short Form Survey subscale “*Energy Fatigue*” also improved on melatonin compared to placebo (U = 53.5; Z = –2.06; *P* = 0.040; ŗ = 0.39). No other differences were found (Supporting Information Tables S4–S6).

Three participants (2 placebo, 1 melatonin) revealed an apnea‐hypopnea index >20 during their second PSG.^22^ However, RBD with dream enactment was observed in these subjects and clearly distinguishable from apnoea‐induced arousals.

Adverse events were mild and included headaches, fatigue, light‐headedness, and morning sleepiness (n = 4 melatonin, n = 5 placebo).^9^ The melatonin dosage of 1 participant was reduced to 2 mg after 3 weeks because of light‐headedness and morning sleepiness, as per protocol. None of the adverse events required further medical attention.

An adherence of 99.0% was found for completing the primary outcome, which remained above 97% for any given week. No associations were found between bedtime variability and treatment responses (Supporting Information).

## Discussion

This small, randomized, placebo‐controlled, double‐blinded, parallel‐group trial indicates that 4 mg of PR melatonin is well tolerated, but not efficacious in ameliorating self‐reported RBD in PD.

These results challenge the current clinical assumptions on the efficacy of melatonin for RBD,^8^, ^9^ although previous studies included small samples with a variety of disorders, questioning their generalizability to PD.^6^, ^7^, ^8^, ^9^, ^11^, ^12^, ^13^, ^14^, ^23^, ^24^, ^25^ The effects of prior trials were often mixed, with several participants not responding to melatonin, even at dosages of up to 25 mg.^14^, ^15^ A discrepancy was also noted between the clinician's subjective impression and objective changes on PSG.^9^, ^13^ Previous findings in uncontrolled and unblinded studies might thus be explained by the robust placebo effect we report here.

Another strength of our study was the use of an RBD‐specific primary outcome (provided in the Supporting Information) that allowed for adequate power by recording daily entries and without assessor bias.^23^

However, the low sample size was not powered to detect group differences on secondary outcomes. The sample size was also too small to definitely rule out an effect of melatonin for reducing RBD. Despite the randomization, the groups differed on the primary outcome at baseline.

The wCIRUS‐RBDQ may not have captured all RBD. This limitation is likely inherent to all studies evaluating RBD, given that patients and their partners are often not aware of the symptoms and there likely exists night‐to‐night variability in frequency and severity.

Not all subjects had bed partners (Supporting Information), which may have limited their ability to notice RBD.^23^ Studies are also encouraged to control for pramipexole intake, which may impact on RBD.^26^ Finally, studies should consider recording actigraphy from the most‐affected arm in PD, given that it may show more RBD.^27^

Importantly, our protocol can now be adopted to test for the efficacy of other RBD treatments, including clonazepam and higher dosages or different formulations of melatonin.^28^, ^29^, ^30^, ^31^, ^32^ Larger, multicentred RCT's are still warranted to assess the efficacy of both drugs for reducing RBD. Such trials should plan for observing strong placebo effects when using patient‐reported outcomes.

In conclusion, administration of melatonin may not be efficacious for reducing symptomatic RBD in PD.

## Author Roles

(1) Research Project: A. Conception and Design; B. Acquisition of Data; C. Analysis and Interpretation of Data; (2) Manuscript: A. Writing of the First Draft, B. Review and Critique; (3) Other: A. Trial coordination, B. Recruitment, C. Led the Protocol and Outcome Development, D. Aided the Protocol Development, E. Created the Randomization Sequence, F. Designed and Performed the Biostatistical Analyses, G. Provided Technical Assistance and Quality Control over the PSG and Actigraphy Data, H. Analyzed the Actigraphy Data, I. Acted as Medical Officer for the Trial Participants, J. Acted as the co‐Principal Investigator, K. Acted as the Head Medical Officer and Principal Investigator.

M.G.: 1B, 1C, 2A, 3A, 3B

A.C.J.: 1A, 2B, 3C

N.S.M.: 2B, 3D, 3E, 3F

D.H.: 1B, 2B

A.E.M.: 2B, 3G

J.M.H.: 1B, 2B

B.A.M.F.: 2B, 3H

B.J.Y.: 1A, 2B, 3I

K.K.H.W.: 1A, 2B, 3I

R.R.G.: 1A, 2B, 3J

S.J.G.L.: 1A, 1B, 2B, 3K

## Financial Disclosures

M.G. was supported by a University of Sydney International Scholarship between 2014 and 2017 and supported by a Postdoctoral Mandate of Internal Funds KU Leuven during manuscript preparation. A.C.J. is supported by the Swiss National Science Foundation (PBGEP3_145338); N.S.M., B.J.Y., K.K.H.W., R.R.G., and S.J.G.L. hold a NeuroSLEEP (AP1060992) grant. R.R.G. is funded by an NHMRC Senior Principal Research Fellowship (1106974). S.J.GL. was supported by an NHMRC Practitioner Fellowship #1003007 and is currently supported by an NHMRC‐ARC Dementia Fellowship #1110414. Neurim Pharmaceuticals Inc. supplied discounted investigational products.

## Supporting information


**Appendix S1**: Supporting Information

**Appendix S2**: Supporting Information

**Figure S1**

**Supplementary Table 1** Inclusion and Exclusion criteria
**Supplementary Table 2**: List of secondary outcome measures
**Supplementary Table 3**: Baseline characteristics of both groups at time of randomisation

**Supplementary Table 4** Secondary polysomnography outcomes
**Supplementary Table 5**: Secondary actigraphy outcomes
**Supplementary Table 6**: Secondary questionnaire outcomes
