# Supplementary material for: Melatonin for rapid eye movement sleep behavior disorder in Parkinson's disease: A randomised controlled trial
Source: Mov Disord. 2019 Oct 31;35(2):344–9. doi: 10.1002/mds.27886 (PMC7027846; doi:10.1002/mds.27886)
Supplement: Supplementary file 1 — Appendix S1: Supporting Information [file MDS-35-344-s001.docx]

**Supplementary information on the Methods and Results**

**Title:** Melatonin for REM sleep behavior disorder in Parkinson’s disease: A randomised controlled trial.

**Authors**: Moran Gilat PhD^1,2,3^, Alessandra Coeytaux Jackson MD^1,4^, Nathaniel S Marshall PhD^1^, Deborah Hammond RN^2^, Anna E Mullins PhD^1^, Julie M Hall MSc^2^, Bernard AM Fang MD^1^, Brendon J Yee MD^1,5^, Keith KH Wong MD^1,5^, Prof Ron R Grunstein MD^1,5^, Prof Simon JG Lewis MD^1,2^.

**Affiliations:**

1. Woolcock Institute for Medical Research, The University of Sydney, Sydney, Australia

2. ForeFront Parkinson’s Disease Research Clinic, Brain and Mind Centre, The University of Sydney, Sydney, Australia

3. Research Group for Neuromotor Rehabilitation, Department of Rehabilitation Sciences, KU Leuven, Leuven, Belgium

4. Department of Neurology, University Hospitals of Geneva, Switzerland

5. Department of Respiratory and Sleep Medicine, Royal Prince Alfred Hospital, Sydney, Australia

**Corresponding author**:

Professor Simon J.G. Lewis

Brain and Mind Centre, University of Sydney, 100 Mallett Street, Camperdown, NSW 2050, Australia

Email: [profsimonlewis@gmail.com](mailto:profsimonlewis@gmail.com)

Telephone: +61 2 9351 0702

**Methods**

*Participants*

We set out to recruit a target sample of 30 people with Parkinson’s disease (PD) and video-PSG confirmed REM sleep behavior disorder (RBD) from specialist clinics at the Woolcock Institute of Medical Research and the Parkinson’s Disease Research Clinic of the Brain and Mind Centre in Sydney, Australia. Participants for this trial were initially recruited on the basis of self- and bed-partner reported symptoms suggestive of RBD. Subsequent eligibility was then assessed using the RBD Screening Questionnaire (RBDSQ) and either a previous video-PSG within last 12 months confirming RBD or a video-PSG within the 4 weeks prior to randomization to confirm the diagnosis.

*Procedures*

The weekly CIRUS-RBD Questionnaire (wCIRUS-RBDQ) contains six items assessing: 1) whether participants took the trial drug; 2) whether they had a bed-partner, and if so, how they spend the night; 3) what time they went to bed; 4) whether they acted out their dreams, and if so, how many times (primary outcome); 5) whether they experienced vivid dreams and if so, how many and how severe were the contents, and; 6) whether or not RBD caused injury to themselves, their bed partners or caused damage to the bedroom environment. A separate wCIRUS-RBDQ was completed for each week of the trial (16 in total).

The protocol included five study visits (Supplementary Figure 1). During visit 1, participants were educated about RBD and shown how to complete the wCIRUS-RBDQ. They were instructed to record any instance of RBD as noted by themselves and/or their bed partners (if applicable). Participants also completed the RBDSQ, the Innsbruck RBD Inventory (RBD-I), and the RBD Questionnaire Hong Kong (RBDQ-HK), which they would later fill out prior to visits 2, 4 and 5. The questionnaires for the screening month also included the Leeds Sleep Evaluation Questionnaire (LSEQ), the Epworth Sleepiness Scale (ESS), the Pittsburgh Sleep Quality Index (PSQI), the Depression and Anxiety Stress Scales (DASS), the 39-item Parkinson’s Disease Questionnaire (PDQ-39), and the 36-item Short Form Survey (SF-36). Participants were instructed to return the wCIRUS-RBDQ’s and questionnaires during each study visit, after which the data was entered into electronic case report forms. Manual entries were quality assured by a second investigator prior to conducting statistical analyses.

During visit 2, a final assessment of eligibility was performed (Supplementary Table 1), followed by the CGI and Movement Disorders Society Unified Parkinson’s Disease Rating Scale (UPDRS). Eligible participants were randomised at the end of visit 2 by providing them with the treatment bottle with their respective randomisation code, which contained the exact amount of study drug required for eight weeks.

During visit 3, a safety assessment was performed. Participants also completed a LSEQ about the first month of treatment.

During visit 4, participants were instructed to return the treatment bottle and a pill count was performed. A blinded assessor performed another CGI and UPDRS. During the second month of treatment the participants again completed the LSEQ, ESS, PSQI, DASS, PDQ-39, and SF-36.

During visit 5, the final wCIRUS-RBDQ’s and questionnaires were returned and another CGI performed. The end of visit 5 concluded the trial for participants. Adverse events were assessed at each study visit and documented on adverse event report forms.

*Secondary objective measures of sleep*

All subjects underwent a baseline PSG within 12 months prior to enrolment, showing sufficient REM sleep without atonia (RSWA) to justify an RBD diagnosis by a sleep physician according to the ICSD-2 diagnostic criteria. Subjects were also asked to wear a Phillips Respironics Actiwatch-II on the wrist least affected by PD and to fill out an associated diary for one week during the baseline period. In the second month of the treatment period, subjects underwent a second PSG and again wore the Actigraphy watch for one week. The actigraphy was scored manually using Phillips Respironics Actiware 5 software. A senior sleep technician visually inspected all PSG’s for data quality and the RSWA index (RSWAI) was calculated from the submentalis EMG signals using an automated algorithm based on the quantitative statistical method of Ferri et al. (2008)^1^.

*Statistical analyses*

Statistical analyses were undertaken by the trial epidemiologist (NSM) or under his direction and supervision.

As the effectiveness of melatonin for RBD may rely on sleep hygiene^2^, a post-hoc analysis was performed to assess whether the outcomes of this trial could have been influenced by variations in bedtimes, as recorded on the wCIRUS-RBDQ for each night of the trial. The groups were compared on their average bedtimes, bedtime variability (SD), and the total sum of bedtime delay and advancement compared to the average bedtime in minutes using independent sample t-tests^3^. Pearson correlation analyses were also performed between these bedtime measures and the primary outcome during weeks 5–8 of treatment in the melatonin group (n=15).

Not all RBD sufferers in the clinical population have a bed-partner. A large proportion of RBD sufferers also spend the night in a different bed or a different room than their partners due to their RBD being disruptive of their partner’s sleep. As the presence of a bed-partner sleeping in the same bed as the patients could not be controlled for over the total duration of the present study, the availability of a bed partner was not set as an *a-priori* exclusion criterion. We argued that if positive effects of melatonin were to be found regardless of the presence of a bed-partner, than the outcomes of this study would have better clinical translation to the home situation of the patients.

**Results**

*Patients*

Supplementary Table 3 details the participants’ baseline characteristics at time of randomisation, including dopaminergic medication intake. Both groups took an approximately equal variety of concomitant pharmacological agents (e.g. statins, anti-inflammatories, laxatives, aspirin) that remained stable during the trial. In the melatonin group, one participant was on an SSRI (sertraline), three on other anti-depressants (2 mirtazapine, 1 amitriptyline), and one on a β1-receptor blocker (metoprolol). In the placebo group, two participants were on an SSRI (1 sertraline, 1 citalopram), one on an SNRI (venlafaxine), and four were on another anti-depressant (mirtazapine). None were taking β-receptor blockers. None of the participants were taking gabapentinoids, such as gabapentin or pregabalin.

*Outcomes*

A sensitivity analysis without the one participant in the melatonin group who scored zero RBD events on the primary outcome (wCIRUS-RBDQ) did not change our conclusions (primary endpoint: 3.6 events/week melatonin vs. 3.7 placebo; difference 0.1; 95%CI=-3.4 to 3.5; p=0.97) and the number of RBD events at baseline only marginally increased in the melatonin group (4.0 instead of 3.4 events/week).

One subject on placebo dropped out prior to the second PSG. Another participant in the placebo group was not willing to undergo a PSG at the end of treatment. As such, the secondary PSG outcomes were analysed between n=15 on melatonin and n=13 on placebo. One participant on melatonin did not experience any REM sleep during the second PSG. The submentalis EMG data of six participants on melatonin and seven on placebo had poor data quality (e.g. impedance >10kΩ) or contained insufficient data during either PSG to reliably calculate the RSWAI. The delta RSWAI was therefore compared between n=8 on melatonin and n=6 on placebo (Supplementary Table 4).

A minimal requirement of five days and nights of actigraphy data was applied. As such, actigraphy could be compared between n=12 on melatonin and n=10 on placebo. The results revealed that the average sleep onset latencies for the melatonin group decreased following treatment compared to a slight increase over time in the placebo group (U=12.0, Z=-3.17, p=0.002, effect size ŗ=0.68). No other group differences were found (all p>0.05, see Supplementary Table 5).

One participant in the melatonin group scored a 2 on the CGI (“*Much improved*”), three scored a 3 (“*Minimally improved*”), and ten scored a 4 (“*No change*”) at the end of treatment (Visit 4). Six participants in the placebo group scored a 3, and eight scored a 4 (two-sided Fisher exact test, p=0.710). No change on the Movement Disorder Society Unified Parkinson’s Disease Rating Scale (UPDRS) was found between groups at the end of treatment compared to screening (median (IQR), Melatonin (n=15): delta (end of treatment - screening) UPDRS total=-4.0 (-24 – 6.0), delta UPDRS-III=-3.0 (-10 – 6.0); Placebo (n=14): delta UPDRS total=-8.0 (-19 – -2), delta UPDRS-III=-6.0 (-8.5 – -3.5); delta UPDRS total: U=77.5, Z=-0.92, p=0.356; delta UPDRS-III: U=84.5, Z=-0.60, p=0.549).

No significant group differences were found on the secondary questionnaires (all p>0.1, see Supplementary Table 6), except for subscale 4 (“*Energy Fatigue*”) on the SF-36, which indicated an improvement in the melatonin group compared to a worsening over time in the placebo group (U=53.5, Z=-2.06, p=0.040, effect size ŗ=0.39).

No significant *post-hoc* differences were found between groups on their averaged bedtime measures (Average bedtime: Melatonin=10:57pm, Placebo=10:47pm, t=0.60, p=0.555; Bedtime variability: Melatonin=54mins, Placebo=46mins, t=1.01, p=0.322; Bedtime delay: Melatonin=269mins, Placebo=252mins, t=0.31, p=0.758; Bedtime advance: Melatonin=207mins, Placebo=143mins, t=1.29, p=0.206). Furthermore, these bedtime measures did not correlate with the primary outcome in the melatonin group (Average bed time: r=0.194, p=0.488; Bed time variability: r=0.304, p=0.271; Bed time delay: r=0.307, p=0.266; Bed time advancement: r=-0.03, p=0.915). Although these *post-hoc* analyses did not reveal associations between bedtime variability and treatment responses in the melatonin group, future trials could utilize actigraphy with sleep diary entries during screening to a-priori exclude participants with poor sleep hygiene^2,4^.

The presence of a bed partner was not an *a-priori* exclusion criterion, and as a result three subjects (2 placebo, 1 melatonin) had no bed partners and four subjects (2 placebo, 2 melatonin) irregularly spent the night with their bed partners. Although this scenario is representative of the RBD population, the lack of a bed partner may have affected the ability of these subjects to notice RBD and accurately complete the wCIRUS-RBDQ. Future studies may wish to include bed-partner availability as an eligibility criterion. The sleep quality of the bed partners was also not assessed. It is likely that good sleeping bed partners would more frequently miss an RBD event. Future studies could be designed to investigate the association between sleep quality of the bed partners and ability to adequately report RBD symptoms.

We also explored the pill counts and participant’s entries on the wCIRUS-RBDQ item 1, which recorded whether or not they took the trial drug for each night. This revealed an overall adherence of 99.8% for filling out this item during the eight-week treatment period. Participants forgot to take the trial drug on average during 3.1% of nights at the primary endpoint. Pill counts were available for 25 out of 29 participants that completed the study, as two participants on placebo and two on melatonin did not return their bottles. Sixteen participants returned <10 tablets, six returned 10-20 tablets and three (2 on melatonin, 1 on placebo) returned >20 tablets. One of the participants in the melatonin group who returned 42 tablets, reported to have never missed a dose, but to have only taken one tablet each night (i.e. 2mg instead of 4mg) during the last weeks of treatment. The two other participants, whom each returned 36 tablets, may have occasionally forgotten to take the trial drug. These three participants were unremarkable, being 54-63 years of age, having an MMSE of 28 or higher, and Hoehn and Yahr stages 1-2.

**References**

1. Ferri R, Manconi M, Plazzi G, et al. A quantitative statistical analysis of the submentalis muscle EMG amplitude during sleep in normal controls and patients with REM sleep behavior disorder. J Sleep Res 2008;17(1):89–100.

2. Kunz D, Mahlberg R. A two-part, double-blind, placebo-controlled trial of exogenous melatonin in REM sleep behaviour disorder. J Sleep Res 2010;19(4):591–596.

3. Taylor BJ, Matthews KA, Hasler BP, et al. Bedtime Variability and Metabolic Health in Midlife Women: The SWAN Sleep Study. Sleep 2016;39(2):457–465.

4. Kunz D. Melatonin in rapid eye movement sleep behavior disorder: why does it work? Sleep Med. 2013;14(8):705–706.
