# Supplementary material for: Melatonin for rapid eye movement sleep behavior disorder in Parkinson's disease: A randomised controlled trial
Source: Mov Disord. 2019 Oct 31;35(2):344–9. doi: 10.1002/mds.27886 (PMC7027846; doi:10.1002/mds.27886)
Supplement: Supplementary file 2 — Appendix S2: Supporting Information [file MDS-35-344-s002.pdf]

WOOLCOCK INSTITUTE *of* MEDICAL RESEARCH

**SUBJECT ID: ………**

# The weekly CIRUS REM sleep behavior disorder questionnaire

*wCIRUS-RBDQ*

Subject ID:

WEEK nr: ☐

**EXPLANATION**

***Please complete this questionnaire <u>with your partner</u>, as you may not recall all the events.*** **ANSWER ALL QUESTIONS.**

The questionnaire covers a 7-night period**.** Please fill in your responses <u>every day and for every event on the following morning,</u> with today's matching date*. If you don't have a partner, any essential and <u>regular observer</u> of your sleep related behavior (other family members, or carer) is considered as a "partner" in this questionnaire.*

This questionnaire will help us assess the severity of your sleep disorder and whether the treatment is working or not.

Your answers to these questions are very important.

***<u>Explanation of terms used over the pages</u>***

***<u>EVENT</u>:*** *Any event such as sleep behavior, dream or injuries that occurred in your sleep <u>once per night</u> (1 event/night) <u>or more</u> (2 to 4 events/night)*

***<u>"VIVID DREAMS"</u>:*** Intense dreams

"**<u>ACTING OUT DREAMS</u>**": *Some patients with REM behavior disorder "act out their dreams" with movements that relate to the content of those dreams, or yelling/talking.. For example, if you are dreaming that you are running away from something, your legs may appear to be running.*

***<u>INJURIES</u>:*** *An injury can include: diving from bed, accidental punches, kicks, bruises, scratches or any damage to the bed environment or any other injuries that required medical intervention*

***<u>MEDICATION:</u>*** *either Circadin (melatonin) or placebo*

## Tips to complete the questionnaire

- Over the page is a diary to be filled out on a daily basis from Monday to Sunday
- There are six main questions: these should be filled out first thing in the morning
- You may experience several dream episodes and several enacting dream behavior events PER NIGHT!
- Please fill in your responses every day and for every event on the following morning, with today's matching date.

**HERE IS AN EXAMPLE OF HOW THE DIARY SHOULD BE FILLED OUT with two events /night.**

For instance, on Monday the subject slept with his/her bed partner in the same room and the same bed, went to bed at 22:30, had two episodes of dream enacting behavior with two episodes of vivid dreams, rated in the order they occurred as the following, unpleasant and horrific. On the first event, the subject fell out of bed without damaging the bed environment. On the second event, the subject broke the window.

| LAST NIGHT | MONDAY | TUESDAY | WEDNESDAY |
|---|---|---|---|
| | _05 /_06 /2013 | _06 /_06 /2013 | _07 /_06 /2013 |
| **1. MEDICATION** I took my medication last night | YES ☑ NO ☐ | YES ☑ NO ☐ | YES ☑ NO ☐ |
| **2. BEDPARTNER:** Do you have a partner? YES ☑ NO ☐ *If NO, go to question 3* | | | |
| **2.1** For each night of the week, describe how you and your bed partner **slept?** **Last night my bed partner was:** | | | |
| **2.2** Same room, same bed | YES ☑ NO ☐ | YES ☐ NO ☐ | YES ☑ NO ☐ |
| **2.3** Same room, different bed | YES ☐ NO ☐ | YES ☐ NO ☐ | YES ☐ NO ☐ |
| **2.4** Different room | YES ☐ NO ☐ | YES ☑ NO ☐ | YES ☐ NO ☐ |
| **2.5** Away from home | YES ☐ NO ☐ | YES ☐ NO ☐ | YES ☐ NO ☐ |
| **3. BEDTIME** Last night I went to bed at __:__ | Bed time 22:30 | Bed time 23:30 | Bed time 22:30 |
| **4. ACTING OUT DREAM** **4.1** Last night I acted out my dream | YES ☑ NO ☐ | YES ☑ NO ☐ | YES ☑ NO ☐ |
| **4.2** How many times did it happen last night? *(write number of episodes last night)* | 2 | 2 | 1 |
| **5. VIVID DREAMS** **5.1** Last night I had vivid dreams | YES ☑ NO ☐ | YES ☑ NO ☐ | YES ☐ NO ☑ |
| **5.2** I describe my vivid dreams | ***0*** ---1---2---3---4--- ***5*** ---6---7---8---9--- ***10*** ☺ ☹ ☹☹ ***0*** *= pleasant, happy dream* ***5*** *= unpleasant* ***10*** *= horrific nightmares* ** Indicate **99** if you don't remember* | | |
| *-Score the quality of your first 4 vivid dreams in order of appearance last night (see chart)* Dream N° | 1: 5, 2: 9, 3: , 4: | 1: 4, 2: 8, 3: , 4: | 1: 9, 2: , 3: , 4: |
| *5.3 More than 4 dreams?* | YES ☐ NO ☑ | YES ☐ NO ☑ | YES ☐ NO ☑ |

| LAST NIGHT | MONDAY | TUESDAY | WEDNESDAY |
|---|---|---|---|
| | 05 /06 /2013 | 06 /06 /2013 | 07/06/2013 |
| **6. INJURY** - Last night, did you do any of the following mentioned below? - Rate the severity of every event according the chart | How severe were those injuries on a scale from 0 to 10? ***Rate EVERY injury, in the order they occurred?*** ***0---1---2---3---4---5---6---7---8---9---1*** ☹ ☹ ☹☹ ***0*** *= WITHOUT ANY CONSEQUENCE* ***5*** *= SEVERE - Injuries that did not require any help from healthcare professional OR/AND minor to mild damage to the bed environment* ***10*** *= VERY SEVERE – Injuries that require medical intervention OR/AND major damage to the bed environment* *Indicate **99** if you don't remember* | | |
| **6.1** Injured MYSELF | YES ☐ NO ☑ | YES ☐ NO ☑ | YES ☑ NO ☐ |
| IF YES, rate the severity — Dream N° | 1: , 2: , 3: , 4: | 1: , 2: , 3: , 4: | 1: , 2: , 3: , 4: |
| **6.2** Injured my BED PARTNER? | YES ☐ NO ☑ | YES ☑ NO ☐ | YES ☑ NO ☐ |
| IF YES, rate the severity — Dream N° | 1: , 2: , 3: , 4: | 1: 2, 2: 5, 3: , 4: | 1: 5, 2: , 3: , 4: |
| **6.3** FELL out of my bed? | YES ☑ NO ☐ | YES ☐ NO ☑ | YES ☐ NO ☑ |
| IF YES, rate the severity — Dream N° | 1: 5, 2: 0, 3: , 4: | 1: , 2: , 3: , 4: | 1: , 2: , 3: , 4: |
| **6.4** Damaged the BED ENVIRONMENT? | YES ☑ NO ☐ | YES ☐ NO ☑ | YES ☑ NO ☐ |
| IF YES, rate the severity — Dream N° | 1: 0, 2: 9, 3: , 4: | 1: , 2: , 3: , 4: | 1: 7, 2: , 3: , 4: |

| LAST NIGHT | MONDAY | TUESDAY | WEDNESDAY | THURSDAY | FRIDAY | SATURDAY | SUNDAY |
|---|---|---|---|---|---|---|---|
| | _ _/_ _/201_ | _ _/_ _/201_ | _ _/_ _/201_ | _ _/_ _/201_ | _ _/_ _/201_ | _ _/_ _/201_ | _ _/_ _/201_ |
| **1. MEDICATION**<br>I took my medication last night | YES ☐ NO ☐ | YES ☐ NO ☐ | YES ☐ NO ☐ | YES ☐ NO ☐ | YES ☐ NO ☐ | YES ☐ NO ☐ | YES ☐ NO ☐ |
| **2. BEDPARTNER:** Do you have a partner? YES ☐ NO ☐ *If **NO**, go to question 3* | | | | | | | |
| **2.1** For each night of the week, describe how you and your bed partner **slept?** *(Please tick all that apply)*<br>**Last night my bed partner was:** | | | | | | | |
| **2.2** Same room, same bed | YES ☐ NO ☐ | YES ☐ NO ☐ | YES ☐ NO ☐ | YES ☐ NO ☐ | YES ☐ NO ☐ | YES ☐ NO ☐ | YES ☐ NO ☐ |
| **2.3** Same room, different bed | YES ☐ NO ☐ | YES ☐ NO ☐ | YES ☐ NO ☐ | YES ☐ NO ☐ | YES ☐ NO ☐ | YES ☐ NO ☐ | YES ☐ NO ☐ |
| **2.4** Different room | YES ☐ NO ☐ | YES ☐ NO ☐ | YES ☐ NO ☐ | YES ☐ NO ☐ | YES ☐ NO ☐ | YES ☐ NO ☐ | YES ☐ NO ☐ |
| **2.5** Away from home | YES ☐ NO ☐ | YES ☐ NO ☐ | YES ☐ NO ☐ | YES ☐ NO ☐ | YES ☐ NO ☐ | YES ☐ NO ☐ | YES ☐ NO ☐ |
| **3. BEDTIME** Last night I went to bed at:_ _:_ _ *(please write time)* | Bed time __:__ | Bed time __:__ | Bed time __:__ | Bed time __:__ | Bed time __:__ | Bed time __:__ | Bed time __:__ |
| **4. ACTING OUT DREAM**<br>**4.** Last night I acted out my dreams | YES ☐ NO ☐ | YES ☐ NO ☐ | YES ☐ NO ☐ | YES ☐ NO ☐ | YES ☐ NO ☐ | YES ☐ NO ☐ | YES ☐ NO ☐ |
| **4.2** How many times did it happen last night?<br>*(write number of episodes last night)* | ☐ | ☐ | ☐ | ☐ | ☐ | ☐ | ☐ |
| **5. VIVID DREAMS**<br>**5.1** Last night I had vivid dreams | YES ☐ NO ☐ | YES ☐ NO ☐ | YES ☐ NO ☐ | YES ☐ NO ☐ | YES ☐ NO ☐ | YES ☐ NO ☐ | YES ☐ NO ☐ |
| **5.2** I describe my vivid dreams as:<br>- Score the quality of your first 4 vivid dreams in order of appearance last night (see chart) | How severe were your dreams?<br>*If you have more than one dream, give a severity rating for each dream*<br>***0*** *--- 1--- 2 --- 3 --- 4 ---* ***5*** *--- 6 --- 7 --- 8 --- 9 ---* ***10***<br>☺ ☹ ☹☹<br>***0*** *= pleasant, happy dream* ***5*** *= unpleasant* ***10*** *= horrific nightmares* *Indicate* ***99*** *if you don't remember the quality of the dream* | | | | | | |
| Dream N°<br>*(Write score 0 to 10 inside boxes for each of the episodes/dreams)* | 1 ☐ 2 ☐ 3 ☐ 4 ☐ | 1 ☐ 2 ☐ 3 ☐ 4 ☐ | 1 ☐ 2 ☐ 3 ☐ 4 ☐ | 1 ☐ 2 ☐ 3 ☐ 4 ☐ | 1 ☐ 2 ☐ 3 ☐ 4 ☐ | 1 ☐ 2 ☐ 3 ☐ 4 ☐ | 1 ☐ 2 ☐ 3 ☐ 4 ☐ |
| **5.3** I had more than 4 dreams? | YES ☐ NO ☐ | YES ☐ NO ☐ | YES ☐ NO ☐ | YES ☐ NO ☐ | YES ☐ NO ☐ | YES ☐ NO ☐ | YES ☐ NO ☐ |

| LAST NIGHT | MONDAY | TUESDAY | WEDNESDAY | THURSDAY | FRIDAY | SATURDAY | SUNDAY |
|---|---|---|---|---|---|---|---|
| | _ _/_ _/201_ | _ _/_ _/201_ | _ _/_ _/201_ | _ _/_ _/201_ | _ _/_ _/201_ | _ _/_ _/201_ | _ _/_ _/201_ |
| **6. INJURY**<br>- Last night, did you do any of the following mentioned below?<br><br>Rate the severity of every event according to the chart<br><br>(*Write score 0 to 10 inside boxes for each of the episodes/dreams)* | How severe were those injuries on a scale from 0 to 10?<br>***Rate EVERY event, in the order they occurred?***<br>***0*** *--- 1--- 2 --- 3 --- 4 ---* ***5*** *--- 6 --- 7 --- 8 --- 9 ---* ***10***<br>☺ ☹ ☹☹<br><br>***0*** *= WITHOUT ANY CONSEQUENCE*<br>***5*** *= SEVERE - Injuries that did not require any help from healthcare professional, minor to mild damage to the bed environment*<br>***10*** *= VERY SEVERE – Injuries that require medical intervention, major damage to the bed environment*<br>*Indicate* ***99*** *if you don't remember* | | | | | | |
| **6.1** Injured MYSELF<br>IF YES, rate the severity<br>Dream N° | YES ☐ NO ☐<br>1 ☐ 2 ☐ 3 ☐ 4 ☐ | YES ☐ NO ☐<br>1 ☐ 2 ☐ 3 ☐ 4 ☐ | YES ☐ NO ☐<br>1 ☐ 2 ☐ 3 ☐ 4 ☐ | YES ☐ NO ☐<br>1 ☐ 2 ☐ 3 ☐ 4 ☐ | YES ☐ NO ☐<br>1 ☐ 2 ☐ 3 ☐ 4 ☐ | YES ☐ NO ☐<br>1 ☐ 2 ☐ 3 ☐ 4 ☐ | YES ☐ NO ☐<br>1 ☐ 2 ☐ 3 ☐ 4 ☐ |
| **6.2** Injured my BED PARTNER?<br>IF YES, rate the severity<br>Dream N° | YES ☐ NO ☐<br>1 ☐ 2 ☐ 3 ☐ 4 ☐ | YES ☐ NO ☐<br>1 ☐ 2 ☐ 3 ☐ 4 ☐ | YES ☐ NO ☐<br>1 ☐ 2 ☐ 3 ☐ 4 ☐ | YES ☐ NO ☐<br>1 ☐ 2 ☐ 3 ☐ 4 ☐ | YES ☐ NO ☐<br>1 ☐ 2 ☐ 3 ☐ 4 ☐ | YES ☐ NO ☐<br>1 ☐ 2 ☐ 3 ☐ 4 ☐ | YES ☐ NO ☐<br>1 ☐ 2 ☐ 3 ☐ 4 ☐ |
| **6.3** FELL out of my bed?<br>IF YES, rate the severity<br>Dream N° | YES ☐ NO ☐<br>1 ☐ 2 ☐ 3 ☐ 4 ☐ | YES ☐ NO ☐<br>1 ☐ 2 ☐ 3 ☐ 4 ☐ | YES ☐ NO ☐<br>1 ☐ 2 ☐ 3 ☐ 4 ☐ | YES ☐ NO ☐<br>1 ☐ 2 ☐ 3 ☐ 4 ☐ | YES ☐ NO ☐<br>1 ☐ 2 ☐ 3 ☐ 4 ☐ | YES ☐ NO ☐<br>1 ☐ 2 ☐ 3 ☐ 4 ☐ | YES ☐ NO ☐<br>1 ☐ 2 ☐ 3 ☐ 4 ☐ |
| **6.4** Damaged the BED ENVIRONMENT?<br>IF YES, rate the severity<br>Dream N° | YES ☐ NO ☐<br>1 ☐ 2 ☐ 3 ☐ 4 ☐ | YES ☐ NO ☐<br>1 ☐ 2 ☐ 3 ☐ 4 ☐ | YES ☐ NO ☐<br>1 ☐ 2 ☐ 3 ☐ 4 ☐ | YES ☐ NO ☐<br>1 ☐ 2 ☐ 3 ☐ 4 ☐ | YES ☐ NO ☐<br>1 ☐ 2 ☐ 3 ☐ 4 ☐ | YES ☐ NO ☐<br>1 ☐ 2 ☐ 3 ☐ 4 ☐ | YES ☐ NO ☐<br>1 ☐ 2 ☐ 3 ☐ 4 ☐ |

**THANK YOU FOR COMPLETING THIS WEEK'S QUESTIONNAIRE!**

CIRUS
Centre for Integrated Research and Understanding of Sleep
