# Supplementary figures and images for: Melatonin for rapid eye movement sleep behavior disorder in Parkinson's disease: A randomised controlled trial
Source: Mov Disord. 2019 Oct 31;35(2):344–9. doi: 10.1002/mds.27886 (PMC7027846; doi:10.1002/mds.27886)

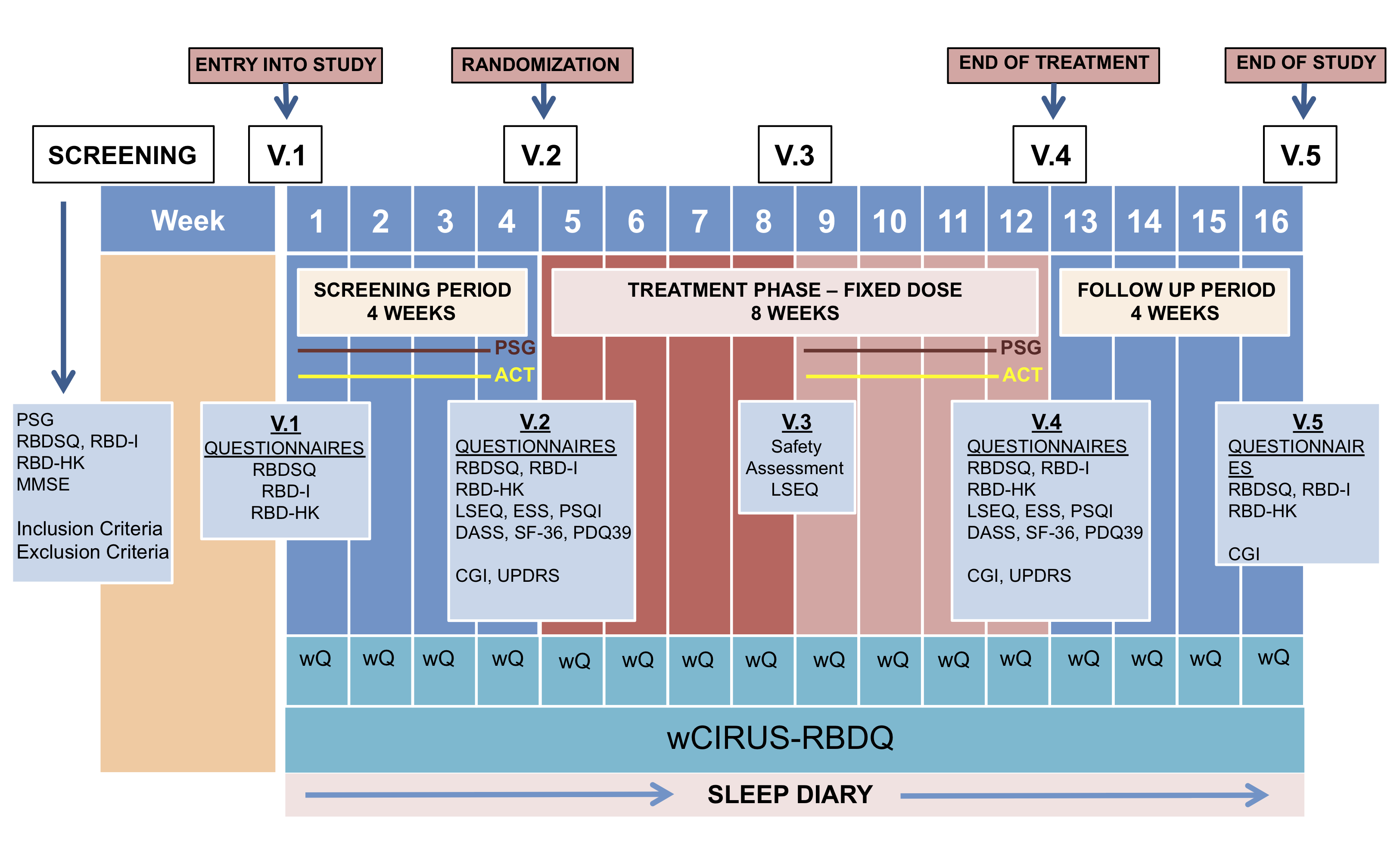

Supplement: Supplementary file 3 — Figure S1 [file MDS-35-344-s003.tiff]
