# Supplementary material for: Melatonin for rapid eye movement sleep behavior disorder in Parkinson's disease: A randomised controlled trial
Source: Mov Disord. 2019 Oct 31;35(2):344–9. doi: 10.1002/mds.27886 (PMC7027846; doi:10.1002/mds.27886)
Supplement: Supplementary file 4 — Supplementary Table 1 Inclusion and Exclusion criteria Supplementary Table 2: List of secondary outcome measures Supplementary Table 3: Baseline characteristics of both groups at time of randomisation [file MDS-35-344-s004.pdf]

Supplementary Table 1: Inclusion and Exclusion criteria

Inclusion criteria

1. Age over 18 years;
2. Mini Mental State Examination (MMSE) ≥ 24;
3. REM sleep behavior disorder (RBD) as defined by the International Classification for Sleep Disorder criteria (ICSD-2);
4. RBD Screening questionnaire criteria (RBDSQ) with a score equal to or greater than 5/13;
5. Two or more events of RBD defined by dream enactment behavior and/or sleep-related injuries (items 2 and 4 of the wCIRUS-RBDQ) during the 4 weeks (screening period) prior to randomisation;
6. Stable medications for 1 month prior to randomisation.

Exclusion criteria

1. Untreated obstructive sleep apnea with an Apnea Hypopnea Index (AHI) >20;
2. Significant psychiatric disorder or cognitive impairment (MMSE< 24);
3. Any significant liver, kidney or autoimmune disease;
4. Current use of benzodiazepines or other non-benzodiazepine hypnotics (e.g. zaleplon, zolpidem, zopiclone), native compounds of melatonin, other melatoninergic agonists, unless washout of more than four weeks;
5. Drug-induced RBD (anti-depressants) with clear relation between RBD onset and medication;
6. Excessive alcohol consumption (>25 Unit/week);
7. Pregnancy or lactation;
8. Use of light therapy (for treatment of sleep-wake disorders) within the last 6 months;
9. Galactose intolerance, LAPP lactase deficiency or glucose-galactose malabsorption.

Supplementary Table 2: List of secondary outcome measures

Secondary outcomes	Time point
1. Mean of the total number of nights per week in which an RBD incident was reported on item 4 of the wCIRUS-RBDQ	Aggregate of nights per week, averaged over weeks 5 to 8 of treatment.
2. Mean of the total number of vivid dreams as reported on item 5 of the wCIRUS-RBDQ	Aggregate of events per week, averaged over weeks 5 to 8 of treatment.
3. Mean of the total number and severity of injuries and bedroom environment damage incidents as reported on item 6 of the wCIRUS-RBDQ	Aggregate of events per week, averaged over weeks 5 to 8 of treatment.
4. REM sleep without atonia index (RSWAI) during video-PSG, as per the method of Ferri et al. (2008).	Improvement in the second PSG at the end of treatment compared to the first PSG at screening.
5. Objective sleep measures as recorded with PSG	Improvement in the second PSG at the end of treatment compared to the first PSG during screening.
6. Objective activity measures during sleep as recorded with actigraphy	Improvement in the second week of actigraphy at the end of treatment compared to the first week of actigraphy during screening.
5. Clinical Global Improvement (CGI) impression scale	At the end of treatment (V.4).
6. Total scores on the RBDSQ, RBD-I, RBD-HK, LSEQ, ESS, PSQI, DASS, PDQ39, and the 8 health related sub-items of the SF-36 questionnaires.	Improvement at the end of treatment (V.4) compared to baseline prior to randomisation (V.2).
7. Parkinson's disease symptom severity as measured with the UPDRS total score and the UPDRS motor symptoms (section 3) sub-score.	Improvement at the end of treatment (V.4) compared to baseline prior to randomisation (V.2).

Abbreviations: RBD=REM sleep Behaviour Disorder; wCIRUS-RBDQ=weekly CIRUS RBD Questionnaire; PSG=Polysomnography; RBDSQ=RBD Screening Questionnaire; RBD-I: Innsbruck RBD Inventory; RBD-HK: RBD Questionnaire Hong Kong; LSEQ: Leeds Sleep Evaluation Questionnaire; ESS: Epworth Sleepiness Scale; PSQI=Pittsburgh Sleep Quality Index; DASS=Depression and Anxiety Stress Scale; PDQ-39=39-item Parkinson's Disease Questionnaire; SF-36=36-item Short Form Survey; V.4=Visit 4 (End treatment); V.2=Visit 2 (Baseline).

Supplementary Table 3: Baseline characteristics of both groups at time of randomisation

	Melatonin (n=15)	Placebo (n=15)
Age (years)	65.3 (6.9)	67.9 (5.3)
Sex (M/F)	12/3	13/2
MMSE	29.3 (1.0)	29.1 (1.2)
Disease duration (years)	5.07 (3.9)	6.13 (4.4)
UPDRS-total	50.8 (28)	55.0 (31)
UPDRS-I	11.1 (5.0)	11.9 (6.9)
UPDRS-II	11.0 (6.9)	10.3 (7.2)
UPDRS-III	27.3 (19)	29.9 (16)
UPDRS-IV	1.40 (1.6)	2.80 (4.1)
Hoehn and Yahr stage#	2 (0*-2.5)	2 (1-2.5)
LEDD	640.1 (310)	621.6 (376)
Number of Participants on: Levodopa monotherapy	2	5
Dopamine agonist monotherapy	0	1
Levodopa + dopamine agonist	2	1
Levodopa + MAO-inhibitor	3	5
MAO-inhibitor monotherapy	1	0
Levodopa + dopamine agonist + MAO-inhibitor	5	2
Levodopa + MAO-inhibitor + COMT-inhibitor	0	1
Dopamine agonist + MAO-inhibitor	1	0
Untreated	1*	0

NOTE: Mean (SD) presented unless otherwise stated. Abbreviations: MMSE = Mini Mental State Examination; UPDRS=Movement Disorder Society Unified Parkinson's Disease Rating Scale; LEDD=Levodopa Equivalent Daily Dose. #Median (range), *Subject with clinically isolated RBD that was diagnosed with PD approximately three months after concluding the study.
