# Supplementary material for: Melatonin for rapid eye movement sleep behavior disorder in Parkinson's disease: A randomised controlled trial
Source: Mov Disord. 2019 Oct 31;35(2):344–9. doi: 10.1002/mds.27886 (PMC7027846; doi:10.1002/mds.27886)
Supplement: Supplementary file 5 — Supplementary Table 4 Secondary polysomnography outcomes Supplementary Table 5: Secondary actigraphy outcomes Supplementary Table 6: Secondary questionnaire outcomes [file MDS-35-344-s005.pdf]

Supplementary Table 4: Secondary polysomnography outcomes

	Melatonin			Placebo				
**Variable**	**Baseline**	**End Treatment**	**Delta (V.4 - V.2)**	**Baseline**	**End Treatment**	**Delta (V.4 - V.2)**	**Test value**	**P value**
**RSWAI**	N=12<br>Median=0.32<br>IQR=0.01 – 0.79	N=9<br>Median=0.51<br>IQR=0.17 – 0.71	N=8<br>Median=-0.056<br>IQR=-0.22 – 0.43	N=11<br>Median=0.50<br>IQR=0.02 – 0.73	N=8<br>Median=0.68<br>IQR=0.30 – 0.85	N=6<br>Median=-0.025<br>IQR=-0.17 – 0.15	U=23.0<br>Z=-0.13	0.897[a]
**Time in Bed (min)**	N=15<br>Mean=426.7<br>SD=48.4	N=15<br>Mean=422.2<br>SD=74.5	N=15<br>Mean=-4.49<br>SD=75.8	N=14<br>Mean=432.1<br>SD=45.0	N=13<br>Mean=447.6<br>SD=50.5	N=13<br>Mean=13.72<br>SD=56.8	T=-0.710	0.484
**Sleep onset Latency (min)**	N=15<br>Median=12.0<br>IQR=2.50 – 33.7	N=15<br>Median=7.80<br>IQR=1.60 – 51.6	N=15<br>Median=0.00<br>IQR=-10.4 – 10.4	N=14<br>Median=11.9<br>IQR=2.85 – 31.3	N=13<br>Median=9.0<br>IQR=1.50 – 17.6	N=13<br>Median=-3.80<br>IQR=-24.6 – 2.75	U=71.5<br>Z=-1.20	0.231 [a]
**REM sleep latency (min)**	N=15<br>Median=186.0<br>IQR=109 – 232	N=15<br>Median=122.1<br>IQR=77.4 – 245	N=15<br>Median=-9.10<br>IQR=-50.5 – 28.1	N=14<br>Median=183.5<br>IQR=141 - 261	N=13<br>Median=145.6<br>IQR=74.9 - 330	N=13<br>Median=-44.6<br>IQR=-100 – 53.2	U=83.0<br>Z=-0.39	0.698 [a]
**Sleep Efficiency (%)**	N=15<br>Median=80.7<br>IQR=71.3 – 88.9	N=15<br>Median=82.6<br>IQR=68.8 – 88.2	N=15<br>Median=2.80<br>IQR=-2.80 – 4.30	N=14<br>Median=80.1<br>IQR=68.4 – 87.1	N=13<br>Median=79.4<br>IQR=73.4 – 88.7	N=13<br>Median=3.50<br>IQR=-10.2 – 9.25	U=90.0<br>Z=-0.346	0.730[a]
**Time awake after sleep onset (min)**	N=15<br>Median=60.5<br>IQR=30.5 – 99.5	N=15<br>Median=57.5<br>IQR=31.7 – 81.1	N=15<br>Median=-6.5<br>IQR=-25.4 – 12.0	N=14<br>Median=73.5<br>IQR=39.4 – 110	N=13<br>Median=90.3<br>IQR=32.4 - 106	N=13<br>Median=-13.1<br>IQR=-24.0 – 42.3	U=94.0<br>Z=-0.161	0.872 [a]
**Total Sleep Time (min)**	N=15<br>Mean=360.6<br>SD=72.4	N=15<br>Mean=353.3<br>SD=69.0	N=15<br>Mean=-7.25<br>SD=88.0	N=14<br>Mean=344.8<br>SD=68.5	N=13<br>Mean=365.2<br>SD=65.6	N=13<br>Mean=17.0<br>SD=73.4	T=-0.784	0.440
**Time in NREM sleep (min)**	N=15<br>Mean=310.0<br>SD=71.9	N=15<br>Mean=291.5<br>SD=54.1	N=15<br>Mean=-18.5<br>SD=75.3	N=14<br>Mean=282.5<br>SD=54.4	N=13<br>Mean=316.7<br>SD=74.0	N=13<br>Mean=31.7<br>SD=58.3	T=-1.95	0.062
**%NREM of total sleep**	N=15<br>Mean=85.9<br>SD=8.0	N=15<br>Mean=85.7<br>SD=9.40	N=15<br>Mean=-0.02<br>SD=12.9	N=14<br>Mean=82.5<br>SD=8.60	N=13<br>Mean=86.2<br>SD=9.50	N=13<br>Mean=3.72<br>SD=8.98	T=-0.917	0.368
**Time in REM sleep (min)**	N=15<br>Mean=50.6<br>SD=29.2	N=15<br>Mean=51.8<br>SD=36.9	N=15<br>Mean=1.17<br>SD=52.5	N=14<br>Mean=62.3<br>SD=36.3	N=13<br>Mean=48.5<br>SD=32.4	N=13<br>Mean=-14.7<br>SD=35.6	T=0.921	0.366
**%REM of total sleep**	N=15<br>Mean=14.1<br>SD=8.0	N=15<br>Mean=14.3<br>SD=9.40	N=15<br>Mean=0.19<br>SD=12.9	N=14<br>Mean=17.5<br>SD=8.60	N=13<br>Mean=13.8<br>SD=9.50	N=13<br>Mean=-3.72<br>SD=8.98	T=0.917	0.368

NOTE: SD=Standard Deviation; IQR=Interquartile Range; [a]=Mann-Whitney U test used to compare the deltas (End of Treatment (V.4) -Baseline (V.2)) between groups due to non-normal data distribution.

Supplementary Table 5: Secondary actigraphy outcomes

	Melatonin			Placebo				
**Variable**	**Baseline**	**End Treatment**	**Delta (V.4 - V.2)**	**Baseline**	**End Treatment**	**Delta (V.4 - V.2)**	**Test Value**	**P Value**
**Sleep onset Latency (min)**	N=12<br>Median=8.67<br>IQR=3.80 – 14.7	N=12<br>Median=7.50<br>IQR=1.52 – 13.5	N=12<br>Median=-2.08<br>IQR=-9.28 – 1.25	N=10<br>Median=2.47<br>IQR=1.86 – 17.4	N=10<br>Median=8.01<br>IQR=4.02 – 32.4	N=10<br>Median=5.66<br>IQR=1.44 – 15.0	U=12.0<br>Z=-3.165	**0.002**
**Total sleep time (min)**	N=12<br>Median=375.6<br>IQR=324 - 448	N=12<br>Median=376.1<br>IQR=354 - 452	N=12<br>Median=20.5<br>IQR=-27.4 – 43.2	N=10<br>Median=405.3<br>IQR=332 - 444	N=10<br>Median=369.2<br>IQR=330 - 404	N=10<br>Median=-11.3<br>IQR=-47.8 – 15.0	U=41<br>Z=-1.25	0.210
**Sleep efficiency**	N=12<br>Median=87.6<br>IQR=80.7 – 91.5	N=12<br>Median=88.3<br>IQR=81.4 – 93.3	N=12<br>Median=1.43<br>IQR=-3.42 – 4.81	N=10<br>Median=86.8<br>IQR=78.5 – 90.6	N=10<br>Median=86.58<br>IQR=77.5 – 90.2	N=10<br>Median=-0.696<br>IQR=-3.04 – 3.65	U=57.0<br>Z=-0.198	0.843
**Wake after sleep onset**	N=12<br>Median=34.0<br>IQR=21.2 – 46.3	N=12<br>Median=34.5<br>IQR=21.2 – 39.4	N=12<br>Median=-1.46<br>IQR=-12.0 – 7.72	N=10<br>Median=39.1<br>IQR=19.4 – 67.0	N=10<br>Median=33.83<br>IQR=21.6 – 51.8	N=10<br>Median=-2.59<br>IQR=-14.0 – 0.18	U=48.0<br>Z=-0.791	0.429
**Sleep activity count**	N=12<br>Median=4150<br>IQR=3063 - 6206	N=12<br>Median=4104<br>IQR=2643 - 4851	N=12<br>Median=-186<br>IQR=-1591 - 910	N=10<br>Median=4778<br>IQR=3012 - 8514	N=10<br>Median=4281<br>IQR=2751 - 6611	N=10<br>Median=-528<br>IQR=-1917 - -90.3	U=47.0<br>Z=-0.857	0.391
**# Naps divided by days of data**	N=12<br>Median=0.32<br>IQR=0.04 – 0.95	N=12<br>Median=0.58<br>IQR=0.02 – 1.45	N=12<br>Median=0.063<br>IQR=-0.11 – 0.61	N=10<br>Median=0.43<br>IQR=0.14 – 1.2	N=10<br>Median=0.46<br>IQR=0.25 – 1.1	N=10<br>Median=0.00<br>IQR=-0.04 – 0.19	U=52.5<br>Z=-0.507	0.612
**Nap duration (min)**	N=12<br>Median=40.6<br>IQR=37.0 – 55.2	N=12<br>Median=45.3<br>IQR=26.4 – 57.8	N=12<br>Median=-3.89<br>IQR=-24.8 – 7.41	N=10<br>Median=34.2<br>IQR=27.4 – 70.2	N=10<br>Median=40.2<br>IQR=25.1 – 44.9	N=10<br>Median=-3.70<br>IQR=-38.7 – 9.39	U=36.0<br>Z<0.001	0.999
**# Sleep bouts**	N=12<br>Median=28.5<br>IQR=19.0 – 34.5	N=12<br>Median=29.8<br>IQR=20.6 – 31.5	N=12<br>Median=0.025<br>IQR=-10.7 – 3.46	N=10<br>Median=26.25<br>IQR=14.8 – 46.7	N=10<br>Median=29.2<br>IQR=22.9 – 35.3	N=10<br>Median=-0.560<br>IQR=-2.73 – 2.96	U=59.0<br>Z=-0.066	0.947
**# Wake bouts**	N=12<br>Median=29.1<br>IQR=22.9 – 33.6	N=12<br>Median=28.7<br>IQR=22.7 – 32.4	N=12<br>Median=-0.06<br>IQR=-8.07 – 1.51	N=10<br>Median=25.8<br>IQR=15.6 – 46.4	N=10<br>Median=29.55<br>IQR=26.5 – 36.9	N=10<br>Median=1.06<br>IQR=-3.73 – 6.53	U=50.0<br>Z=-0.659	0.510
**Wake activity count**	N=12<br>Median=138631<br>IQR=109684 - 167374	N=12<br>Median=142762<br>IQR=104799 - 195075	N=12<br>Median=-2963<br>IQR=-22949 – 32057	N=10<br>Median=162393<br>IQR=112480 - 185453	N=10<br>Median=185309<br>IQR=152189 - 247495	N=10<br>Median=22915<br>IQR=4642 - 56730	U=31<br>Z=-1.91	0.056
**Fragmen-tation Index (FI)**	N=12<br>Median=28.5<br>IQR=25.2 – 35.6	N=12<br>Median=32.6<br>IQR=22.4 – 37.4	N=12<br>Median=0.08<br>IQR=-4.63 – 6.04	N=10<br>Median=29.5<br>IQR=23.0 – 46.1	N=10<br>Median=28.9<br>IQR=24.8 – 41.9	N=10<br>Median=-0.40<br>IQR=-10.3 – 1.63	U=46<br>Z=-0.923	0.356

NOTE: Mann-Whitney U test used to compare the deltas (End of Treatment (V.4) - Baseline (V.2)) between groups due to non-normal data distribution. IQR=Interquartile Range

Supplementary Table 6: Secondary questionnaire outcomes.

	Melatonin			Placebo				
Variable	Baseline	End Treatment	Delta (V.4 - V.2)	Baseline	End Treatment	Delta (V.4 - V2.)	Test Value	P Value
**RBDSQ**	N=15<br>Median=10.0<br>IQR=8.0 - 11.0	N=15<br>Median=9.00<br>IQR=7.0 - 10.0	N=15<br>Median=0.00<br>IQR=-2.0 - 1.0	N=15<br>Median=11.0<br>IQR=10.0 – 12.0	N=14<br>Median=9.50<br>IQR=6.75 – 12.0	N=14<br>Median=0.00<br>IQR=-3.0 – 0.25	U=86.0<br>Z=-0.842	0.400
**RBD-I**	N=15<br>Median=1.00<br>IQR=0.75 – 1.0	N=15<br>Median=0.800<br>IQR=0.40 – 1.0	N=15<br>Median=0.00<br>IQR=-0.20 – 0.0	N=15<br>Median=0.80<br>IQR=0.60 – 1.0	N=14<br>Median=0.78<br>IQR=0.67 – 1.0	N=14<br>Median=0.00<br>IQR=-0.25 – 0.25	U=81.0<br>Z=-1.08	0.281
**RBD-HK**	N=15<br>Median=40.0<br>IQR=20.0 – 48.0	N=15<br>Median=34.0<br>IQR=19.0 – 44.0	N=15<br>Median=-4.00<br>IQR=-12.0 – 7.0	N=15<br>Median=45.0<br>IQR=33.0 – 50.0	N=13<br>Median=34.0<br>IQR=25.5 – 42.5	N=13<br>Median=-10.0<br>IQR=-16.0 - -0.50	U=73.0<br>Z=-1.13	0.258
**PSQI**	N=15<br>Median=6.00<br>IQR=5.0 – 7.0	N=15<br>Median=7.00<br>IQR=4.0 – 8.0	N=15<br>Median=0.00<br>IQR=-1.0 – 2.0	N=15<br>Median=6.00<br>IQR=4.0 – 11.0	N=14<br>Median=6.50<br>IQR=4.0 – 8.0	N=14<br>Median=-0.5<br>IQR=-3.25 – 1.0	U=74.0<br>Z=-1.37	0.171
**LEEDS**	N=14<br>Median=45.8<br>IQR=37.3 – 52.1	N=15<br>Median=46.0<br>IQR=38.3 – 50.1	N=14<br>Median=2.94<br>IQR=-5.5 – 8.41	N=14<br>Median=41.5<br>IQR=32.5 – 51.3	N=14<br>Median=55.1<br>IQR=50.2 – 59.5	N=14<br>Median=9.75<br>IQR=-0.44 – 20.9	U=59.0<br>Z=-1.55	0.120
**ESS**	N=14<br>Median=9.00<br>IQR=3.75 – 12.8	N=13<br>Median=11.0<br>IQR=6.0 – 12.5	N=12<br>Median=0.00<br>IQR=-1.0 – 0.75	N=15<br>Median=8.0<br>IQR=3.0 -11.0	N=14<br>Median=6.50<br>IQR=2.0 – 12.5	N=14<br>Median=-0.5<br>IQR=-2.25 – 2.25	U=76.0<br>Z=-0.418	0.676
**DASS**	N=15<br>Median=17.0<br>IQR=6.0 – 27.0	N=15<br>Median=22.0<br>IQR=17.0 – 32.0	N=15<br>Median=3.00<br>IQR=-2.0 – 9.0	N=15<br>Median=15.0<br>IQR=8.0 – 32.0	N=14<br>Median=20.0<br>IQR=5.0 – 35.8	N=14<br>Median=0.50<br>IQR=-6.0 – 6.75	U=85.0<br>Z=-0.874	0.382
**PDQ-39**	N=14<br>Median=32.0<br>IQR=20.8 – 66.3	N=14<br>Median=36.0<br>IQR=28.5 – 63.0	N=13<br>Median=4.00<br>IQR=-4.5 – 12.0	N=15<br>Median=27.0<br>IQR=19.0 –50.0	N=14<br>Median=32.0<br>IQR=14.5 – 48.5	N=14<br>Median=-0.50<br>IQR=-10.0 – 4.50	U=61.5<br>Z=-1.43	0.152
**SF-36**:<br>1. Physical functioning	N=14<br>Median=72.5<br>IQR=53.8 – 81.3	N=15<br>Median=70.0<br>IQR=45.0 – 75.0	N=14<br>Median=-5.00<br>IQR=-21.3 – 10.0	N=15<br>Median=70.0<br>IQR=60.0 – 80.0	N=14<br>Median=60.0<br>IQR=45.0 – 76.3	N=14<br>Median=0.00<br>IQR=-16.3 – 10.0	U=87<br>Z=-0.509	0.611
**SF-36**:<br>2. Limitations physical health	N=14<br>Median=37.5<br>IQR=0.00 – 100.0	N=15<br>Median=25.0<br>IQR=25.0 – 100.0	N=14<br>Median=0.00<br>IQR=-6.25 – 25.0	N=15<br>Median=25.0<br>IQR=0.00 – 100.0	N=14<br>Median=50.0<br>IQR=0.00 – 100.0	N=14<br>Median=0.00<br>IQR=0.00 – 50.0	U=85.5<br>Z=-0.615	0.538

**SF-36**: 3. Limitations emotional problems	N=14 Median=100.0 IQR=0.00 – 100.0	N=15 Median=100.0 IQR=33.3 – 100.0	N=14 Median=0.00 IQR=-33.3 – 0.00	N=15 Median=100.0 IQR=33.3 – 100.0	N=14 Median=83.3 IQR=33.3 – 100.0	N=14 Median=0.00 IQR=-33.3 – 33.3	U=94.5 Z=-0.173	0.863
**SF-36**: 4. Energy Fatigue	N=14 Median=45.0 IQR=30.0 – 55.0	N=15 Median=50.0 IQR=25.0 – 60.0	N=14 Median=7.50 IQR=-6.25 – 12.5	N=15 Median=60.0 IQR=45.0 – 75.0	N=14 Median=52.5 IQR=33.8 – 65.0	N=14 Median=-10.0 IQR=-15.0 - 0.00	U=53.5 Z=-2.06	**0.040**[1]
**SF-36**: 5. Emotional Wellbeing	N=14 Median=80.0 IQR=64.0 – 88.0	N=15 Median=68.0 IQR=52.0 – 84.0	N=14 Median=-8.00 IQR=-17.0 – 0.00	N=15 Median=80.0 IQR=72.0 – 88.0	N=14 Median=70.0 IQR=56.0 – 83.0	N=14 Median=-8.00 IQR=-20.0 – 3.0	U=93.5 Z=-0.208	0.835
**SF-36**: 6. Social Functioning	N=14 Median=81.3 IQR=50.0 – 100.0	N=15 Median=87.5 IQR=50.0 – 87.5	N=14 Median=0.00 IQR=-15.6 – 12.5	N=15 Median=75.0 IQR=75.0 – 87.5	N=14 Median=75.0 IQR=59.4 – 78.1	N=14 Median=-6.25 IQR=-15.6 – 0.00	U=87.0 Z=-0.521	0.602
**SF-36**: 7. Pain	N=14 Median=67.5 IQR=45.0 – 90.0	N=15 Median=67.5 IQR=45.0 – 77.5	N=14 Median=0.00 IQR=-3.13 – 13.1	N=15 Median=67.5 IQR=55.0 – 80.0	N=14 Median=57.5 IQR=45.0 – 90.0	N=14 Median=0.00 IQR=-10.0 – 10.6	U=95.0 Z=-0.141	0.888
**SF-36**: 8. General Health	N=14 Median=45.0 IQR=30.0 – 66.3	N=15 Median=60.0 IQR=35.0 – 75.0	N=14 Median=7.50 IQR=-5.0 – 15.0	N=15 Median=55.0 IQR=40.0 – 65.0	N=14 Median=45.0 IQR=30.0 – 65.0	N=14 Median=-2.50 IQR=-17.5 – 6.30	U=67.5 Z=-1.41	0.159

NOTE: Group comparison performed on the delta's (End of Treatment (V.4) - Baseline (V.2)) using a Mann-Whitney U test due to non-normal data distribution; IQR=Interquartile Range; The group comparisons on the delta's could only be performed on the subjects that had no missing data for both V.2 and V.4, the median delta values may therefore not match the medians as shown for V.2 and V.4 separately; [1]=Higher scores on the SF-36 indicate a more favorable health state.

List of abbreviations: V.2=Visit 2 at baseline prior to randomisation; V.4=Visit 4 at end of treatment period; RSWAI = REM Sleep Without Atonia Index; (N)REM= (Non) Rapid Eye Movement sleep; %REM=percentage of time spent in REM sleep compared to total sleep duration; IQR=Interquartile range; SD=Standard Deviation; RBDSQ=REM sleep Behavior Disorder Screening Questionnaire; RBD-I=Innsbruck RBD Inventory; RBD-HK=RBD questionnaire Hong Kong; PSQI=Pittsburgh Sleep Quality Index; LEEDS=Leeds Sleep Evaluation Questionnaire; ESS=Epworth Sleepiness Scale; DASS=Depression and Anxiety Stress Scale; PDQ-39=39-item Parkinson's Disease Questionnaire; SF-36=36-item Short Form Survey.
